# In Vitro and In Silico Evaluation of Bikaverin as a Potent Inhibitor of Human Protein Kinase CK2

**DOI:** 10.3390/molecules24071380

**Published:** 2019-04-08

**Authors:** Samer Haidar, Dagmar Aichele, Robin Birus, Janine Hielscher, Tuomo Laitinen, Antti Poso, Joachim Jose

**Affiliations:** 1 Institut für Pharmazeutische und Medizinische Chemie, PharmaCampus, Westfälische Wilhelms-Universität Münster, 48149 Münster, Germany; dagmar.aichele@uni-muenster.de (D.A.); robin.birus@uni-muenster.de (R.B.); j_hiel02@uni-muenster.de (J.H.); joachim.jose@uni-muenster.de (J.J.); 2 Faculty of Pharmacy, Damascus University, Damascus P.O. Box 9411, Syria; 3 School of Pharmacy, Faculty of Health Sciences, University of Eastern, 70211 Kuopio, Finland; tuomo.laitinen@uef.fi (T.L.); antti.poso@uef.fi (A.P.)

**Keywords:** antiproliferation, bikaverin, cell viability, molecular dynamics, permeability

## Abstract

Protein kinase CK2 is an emerging target for therapeutic intervention in human diseases, particularly in cancer. Inhibitors of this enzyme are currently in clinical trials, indicating the druggability of human CK2. By virtual screening of the ZINC database, we found that the natural compound bikaverin can fit well in the ATP binding site of the target enzyme CK2. By further in vitro evaluation using CK2 holoenzyme, bikaverin turned to be a potent inhibitor with an IC_50_ value of 1.24 µM. In this work, the cell permeability of bikaverin was determined using a Caco-2 cell permeability assay as a prerequisite for cellular evaluation and the compound turned out to be cell permeable with a P_app_- value of 4.46 × 10^−6^ cm/s. Bikaverin was tested for its effect on cell viability using a MTT assay and cell proliferation using an EdU assay in different cancer cell lines (MCF7, A427 and A431 cells). Cell viability and cell proliferation were reduced dramatically after treatment with 10 µM bikaverin for 24 h. Additionally the IncuCyte^®^ live-cell imaging system was applied for monitoring the cytotoxicity of bikaverin in the three tested cancer cell lines. Finally, molecular dynamic studies were performed to clarify the ligand binding mode of bikaverin at the ATP binding site of CK2 and to identify the amino acids involved.

## 1. Introduction

**Figure 1:**
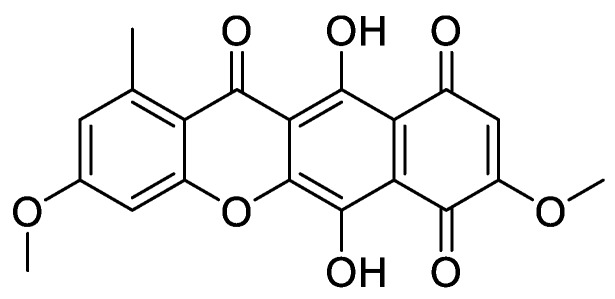
Bikaverin (6,11-dihydroxy-3,8-dimethoxy1-methylbenzo[b]xanthene-7,10,12-trione), a natural compound isolated from *Fusarium vasinfectum* that shows inhibitory activity toward CK2 [9].

Protein kinase CK2 is a ubiquitously expressed serine/threonine kinase with a broad spectrum of substrates. It is composed of two regulatory β-subunits and two catalytic α or α’-subunits. This enzyme plays an important role in several cellular processes such as regulation of the cell cycle and proliferation, beside the suppression of apoptosis. CK2 plays a role in the development of several diseases such as cancer [1], virus infection [2], multiple sclerosis [3], cardiac hypotrophy [4], and others. The inhibition of CK2 can lead to cell apoptosis and tumor cell death, making it an attractive anticancer target and a large number of inhibitors was developed for this enzyme during the last 30 years [1,5]. Most of these compounds are ATP competitive inhibitors and one of them has reached clinical study phase II (the benzonaphthyridine derivative CX-4945, silmitasertib). Silmitasertib is evaluated together with gemcitabine and cisplatin for the treatment of several solid cancers such as cholangiocarcinoma [6,7]. Different CK2 inhibitors were discovered using computer-aided drug design (CADD), such as the well-known inhibitors ellagic acid and quinalizarin [6]. Additionally, it is important to mention that several inhibitors for this enzyme are from natural sources, such as emodin, ellagic acid and others [8,9]. It might be relevant to note that natural compounds are important sources for drug development. According to the World Health Organization, more than 21,000 plant species are used in herbal medicine [10]. According to Newman et al. [11], the natural products field is producing or is involved in around 50% of the small molecules approved as drugs. These compounds are either natural, botanical or naturally-derived semi-synthetic modified compounds. The high number of natural or naturally-derived compounds reflects the importance of these compounds as a source in the drug pipeline. With our ongoing attempts to search for highly active CK2 inhibitors, we were recently able to develop a pharmacophore model depending on a series of indeno[1,2-*b*]indoles [9]. This model was used to search the “all now” part of the ZINC database [12], which contains around 13 million commercially available compounds. Out of these compounds, 55 candidates were selected from among the best sorted candidates using a scoring function and visual inspection by MOE software v2016.10 (Molecular Operating Environment (MOE), C.C.G.I., Montreal, QC, Canada) [13]. Using this method, bikaverin was found (Figure 1).

Bikaverin, also known as Lycopersin, was isolated around seventy years ago [14] from cultures of *Fusarium lycopersici* and *Fusarium vasinfectum*; later on, it was isolated from other fungal species [15]. This reddish pigment has gained attention because it showed antibiotic and antitumoral properties as well as hemolytic effects [15,16]. During the last two decades, several reports were published focusing on the synthesis, production and properties of bikaverin and were reviewed as well [15,17,18,19]. Only a few reports focused on the mode of action of bikaverin, and no inhibition of CK2 was reported. For this reason, and since this inhibitor is a natural compound with antitumor activity, we focused on its in vitro effect, its cellular penetration as well as its effect on different cancer cells. Previously, our in vitro results showed that bikaverin inhibited the target enzyme when tested on purified human protein kinase CK2 expressed in *Escherichia coli* with an IC_50_ value of 1.24 µM [9]. Here we report on the effect of bikaverin on cell viability and the anti-proliferative effect on MCF7, A427 and A431 cells. The ability of bikaverin to penetrate the cell membrane together with two other known inhibitors of CK2 was determined first using an in vitro Caco-2 cell culture model (human epithelial colorectal adenocarcinoma cells). In addition, a molecular dynamic study was performed to probe the stability of the ligand binding mode.

## 2. Results and Discussion

**Figure 2:**
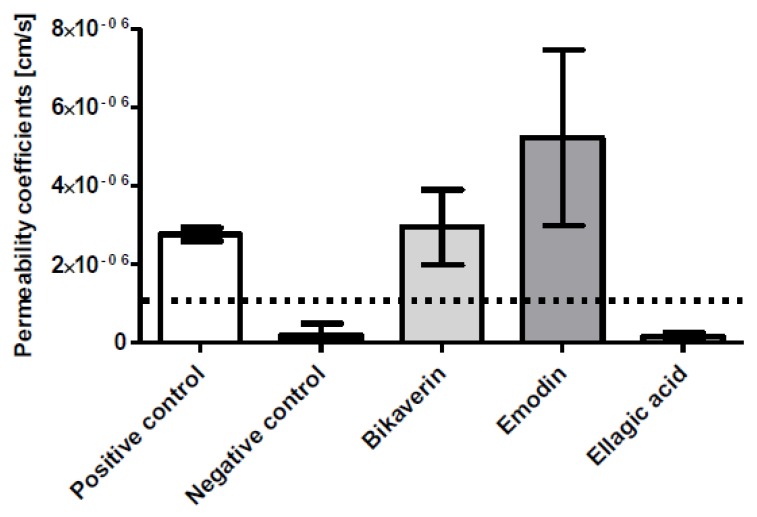
Cell permeability of bikaverin compared to emodin and ellagic acid using a Caco-2 assay based on human epithelial colorectal adenocarcinoma cells. Rhodamine B served as a positive control and FITC-dextran 4 as a negative control. The dotted line indicates the P_app_ limit for a drug that is supposed to be completely absorbed.

A prerequisite of the cellular effects of bikaverin is to determine its cell permeability, so an in vitro model for the direct determination of the permeability coefficient was used in this study. For this purpose, the Caco-2 cell permeability assay is the most common tool [20,21]. Here, Caco-2 cells were used to elucidate the cell membrane permeability of bikaverin and to compare it with two known natural CK2 inhibitors: emodin and ellagic acid. The cell permeability coefficients P_app_- value for bikaverin was determined to be 2.89 × 10^−6^ cm/s, which was almost five times higher than the P_app_- value of FITC-labeled dextran-4 (9.71 × 10^−7^ cm/s), a standard control representing a non-permeable compound. The P_app_- value of bikaverin was in the same range as that obtained for the internal positive control rhodamine B (which is a known membrane permeable florescence dye for living cells [22]) with a P_app_- value of 2.71 × 10^–6^ cm/s, which strongly supported the cell permeability of bikaverin. It is clear from Figure 2 and Table 1 that bikaverin is permitted through the human cell membrane. The cell permeability of emodin (P_app_- value of 5.15 × 10^−6^ cm/s) appeared to be similar to that of bikaverin, whereas that of ellagic acid appeared to be much lower (P_app_- value of 0.16 × 10^−6^ cm/s). The cell permeability of ellagic acid has been investigated before and found to have a P_app_- value of 0.347 × 10^−6^ cm/s, which is in consensus with our results [21]. It is important to note that completely absorbed drugs usually show a P_app_ > 1 × 10^−6^ cm/s [23].

**Figure 3:**
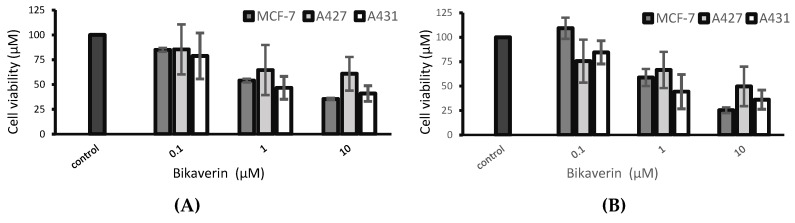
Cell viability of MCF7, A427 and A431 cells after (**A**) 24 h and (**B**) 48 h treatment with bikaverin at different concentrations as tested by MTT assay. Values are given as mean ± SD, *n* = 3.

**Figure 4:**
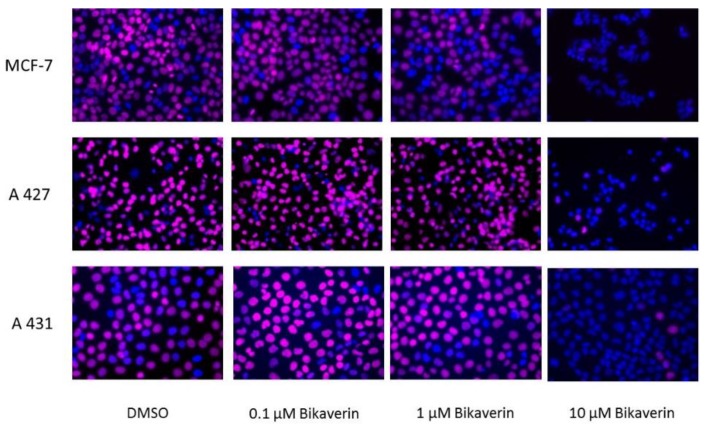
Fluorescence images of MCF7, A427 and A431 cells treated with different concentrations of bikaverin for 24 h. Cell nuclei were double stained by Hoechst 33342 (blue fluorescence), and by EdU-assay using 5-TAMRA-PEG3-azide as a coupled fluorophore (violet fluorescence). Proliferating cells were monitored by EdU-assay. The pictures are overlay of the fluorescence images of Hoechst-stained cells and TAMRA-labeled proliferating cells. The cells that are emitting only blue fluorescence are not proliferating, in contrast to those emitting an additional violet fluorescence. A Keyence microscope was used to obtain the pictures using a 40-fold lens.

**Figure 5:**
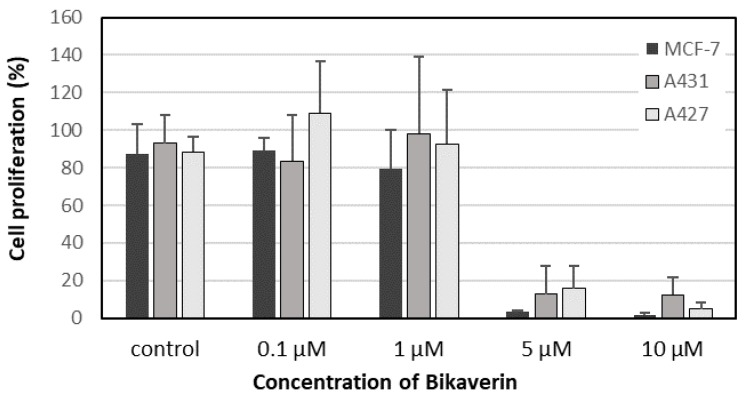
Quantification of the antiproliferative effect of bikaverin using EdU assay on MCF7 (Black), A431 (Dark grey) and A427 (Light grey) cells after 24 h of incubation. Results are shown as percent of proliferating cells relative to control cells (with 1% DMSO) and represent the mean (± SD) of three independent experiments.

**Figure 6:**
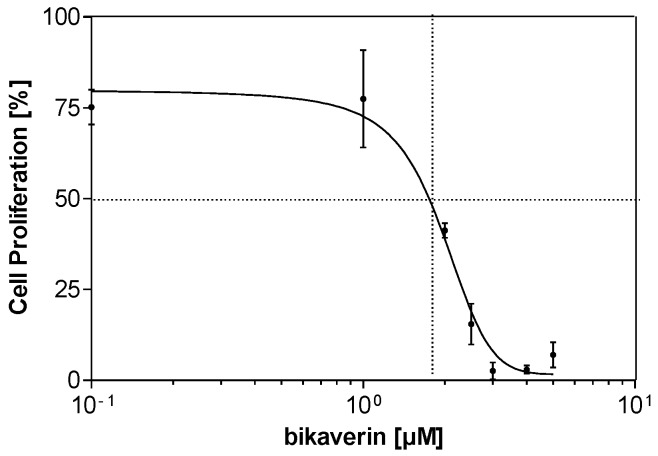
Dose dependent inhibition of MCF7 cell proliferation by bikaverin using EdU assay. The EC_50_ is 1.97 µM, which was determined by three independent replications and mean values with corresponding standard deviations are given.

In order to evaluate the anticancer effect of bikaverin, MCF7 (human breast adenocarcinoma cell line), A427 (human lung carcinoma cell line) and A431 (human epidermoid carcinoma cell line) cells were treated with different concentrations of the compound. The three cell lines were selected as a model because they have been reported to overexpress CK2 and several CK2 inhibitors showed effect on these cancer cells [25,26,27]. Cell viability was tested by applying a MTT assay using the three above mentioned cell lines. As shown in Figure 3A, bikaverin reduced the cell viability of MCF7, A427 and A431 cells to around 45%, 35% and 55%, respectively after 24 h of incubation at a concentration of 1 µM. At a concentration of 10 µM, the compound reduced cell viability to 65%, 40% and 60% for MCF7, A427 and A431, respectively. A prolonged incubation time up to 48 h reduced the cell viability by 5–10% more for all cell lines as shown in Figure 3B. It is relevant to note that emodin was not able to reduce the cell viability in MCF-7 cells at concentration of 10 µM and only 50% of cell viability was reduced at a concentration of 30 µM (data not shown). Additionally, the antiproliferative effect of bikaverin was investigated with the three cell lines mentioned above. For this purpose, a commercially available EdU-click assay was applied, resulting in the coupling of a TAMRA fluorophore into the nucleic acid of cells performing DNA replication and hence preparing cell division [28]. Proliferating cells can be recognized by a violet fluorescence of their nuclei (Figure 4). The number of proliferating cells was determined after treatment with bikaverin in various concentrations and was set into relation to the total number of cells obtained with the 1% DMSO control. As shown in Figure 5, only 2% of MCF7 cells, 5% of A427 cells, and 12% of A431 cells were still able to synthesize DNA upon incubation with 10 μM bikaverin for 24 h. After incubation of MCF7 cells for 48 h with 10 µM bikaverin less than 1% of the cells were still proliferating (data not shown). The EC_50_ of bikaverin (24 h) was determined for MCF7, A427 and A431 cells and appeared to be 1.97 µM, 2.81 µM and 3.27 µM, respectively. The dose response curve as used for EC_50_ value determination is exemplified for MCF7 in Figure 6. Staining the cells with Hoechst 33342, (2′-(4-ethoxyphenyl)-5-(4-methyl-1-piperazinyl)-2,5′-bi-1*H*-benzimidazole trihydrochloride) indicated significant reduction in the total cell number after treatment of the three cell lines with 10 µM bikaverin (Figure 4).

**Figure 7:**
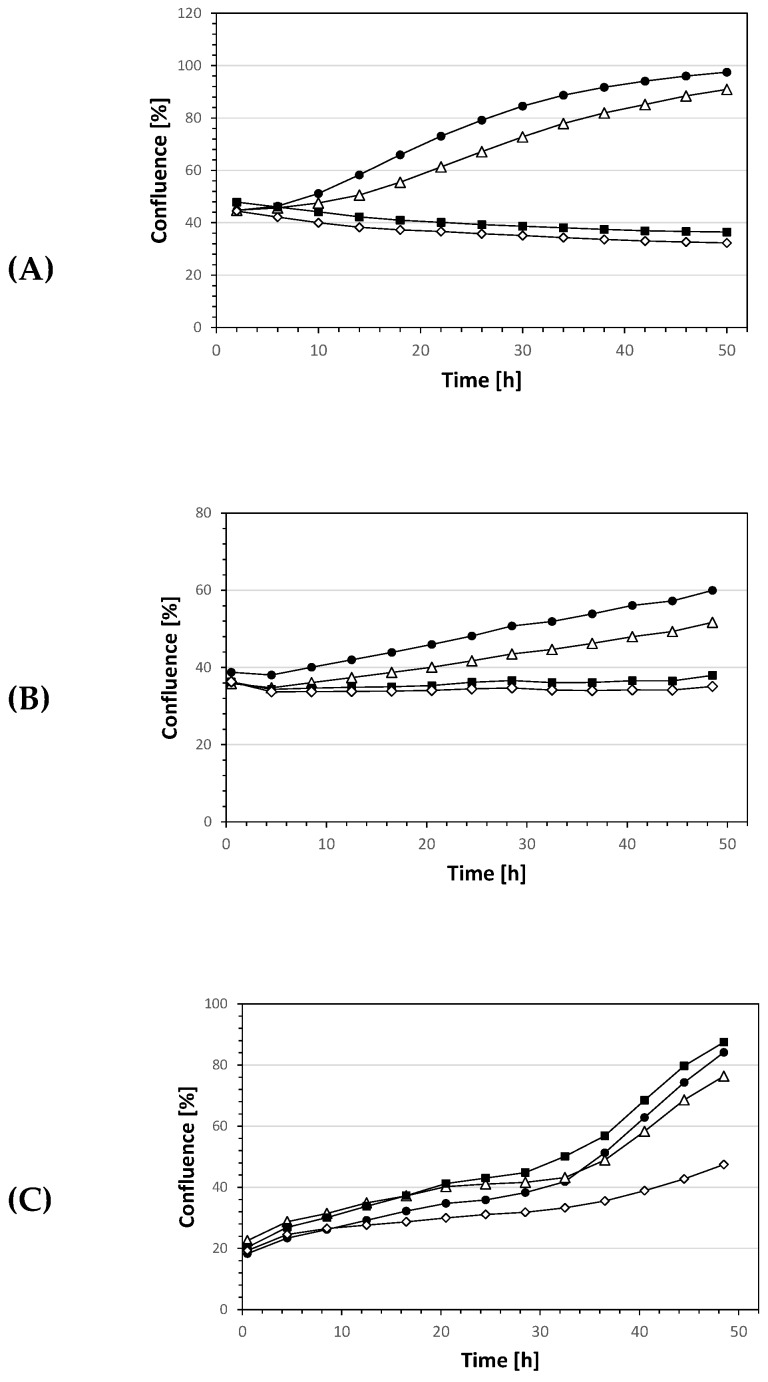
IncuCyte^®^ cell proliferation assay with different concentrations of bikaverin used for treating (**A**) MCF7 cells (**B**) A427 cells (**C**) A431 cells. DMSO 1% (black circle), 1 µM (triangle), 5 µM (black square), 10 µM (prism).

As a further parameter, inhibition of cell growth was evaluated by live cell monitoring using the IncuCyte^®^ S3 live-cell analysis system (Sartorius, Michigan, MI, USA) to determine the confluence after treatment with bikaverin. Cell growth of MCF7 cells was completely blocked after the addition of 5 µM or 10 µM bikaverin, whereas the confluence of the cells after the addition of 1 µM bikaverin was comparable to the control. The confluence of the control increased from 44.7% at the beginning of treatment to 97.5% after incubation for 48 h (Figure 7) and around 1.5% per hour within the linear range between 14 h and 34 h. In contrast, treatment of cells with 10 µM bikaverin led to a decrease in confluence of 0.9% per hour. A similar pattern was seen for the effect of bikaverin on the cell growth of A427 which was completely blocked after treatment with 10 µM bikaverin, while A431 cells showed a slower growth in comparison to the control when treated with 10 µM of the compound as shown in Figure 7. As a matter of fact, A431 cells treated with 10 µM bikaverin reached a confluence of only 44% after 48 h of incubation, while control cells treated with DMSO showed up to 83% confluence. It is important to note that the three cell lines were treated in the same manner and were seeded after 24 h. Nevertheless, the growth of A431 cells was slower in comparison to MCF7 and A427 cells which can explain the differences in the confluence of A431 cells.

**Figure 8:**
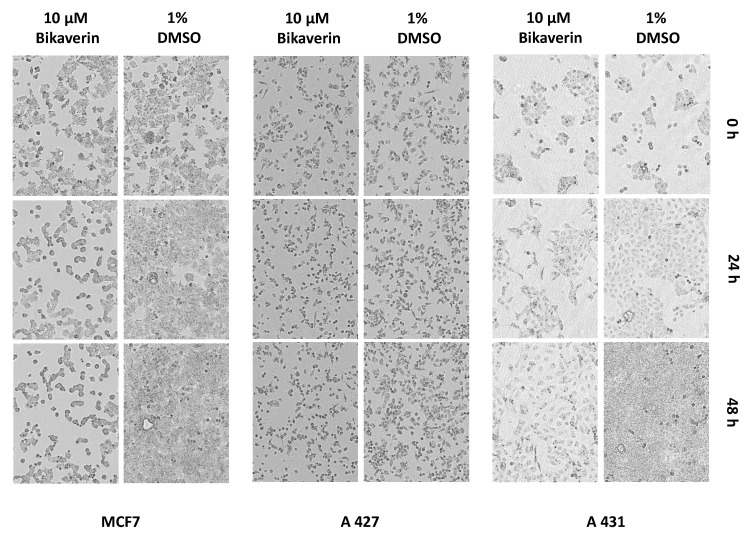
MCF7, A427 and A431 cells treated with 1% DMSO or with 10µM bikaverin for 0h, 24 h and 48 h and recorded by the IncuCyte^®^ live cell imager. The pictures were obtained using the IncuCyte^®^ live cell imager with a ten-fold lens.

In Figure 8, the morphology of MCF7, A427 and A431 cells after treatment with 10 µM of bikaverin for 24 h and 48 h is shown. Cells treated with 1% DMSO were used as a control and the morphology was recorded by the IncuCyte^®^ live cell imager. It is obvious that the number of the cells as well as their shape were changed dramatically using 10 µM of bikaverin for 24 h and 48 h in case of MCF7 and A427. A lesser effect is shown for A431.

**Figure 9:**
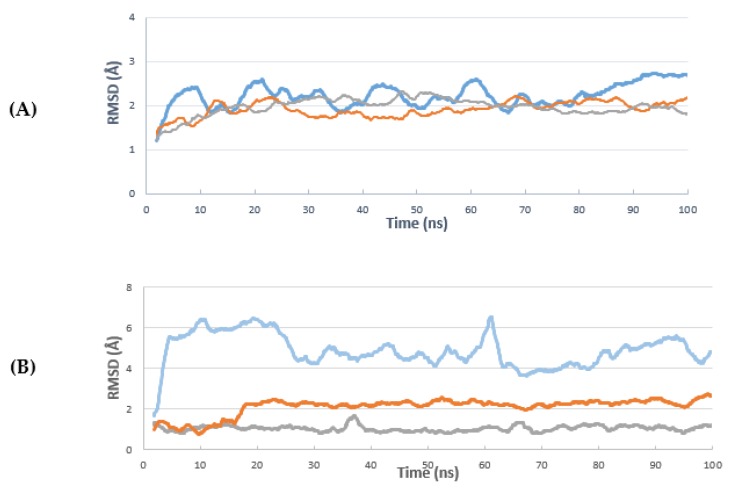
The root-mean-square deviation (RMSD) curve (**A**) curve of the deviations complex with bikaverin (blue line), ellagic acid (brown line), and emodin (gray line). RMSD indicates root-mean-square deviation protein C-alpha atoms, where the moving average over 20 frames is shown for clarity. (**B**) The ligand RMSD curves centered to protein, bikaverin (blue line), ellagic acid (brown line), and emodin (gray line). RMSD indicates root-mean-square deviation of ligand heavy atoms, where the moving average over 20 frames is shown for clarity.

Molecular dynamics (MD) simulation can be used to predict the stability, movement, and conformation of protein–ligand complexes which are observed over comparable time periods. Unlike molecular docking, which neglects the flexibility of proteins and regards them as relatively rigid structures, MD simulations take into account the conformational flexibility and atomic-level dynamics of the proteins which play an essential role in protein functions. With the aim of determining the stability of bikaverin in the active site of the CK2 holoenzyme, a molecular dynamic study was performed using Desmond Molecular Dynamics System (Schrödinger). The same was done for ellagic acid and emodin and the results were compared. A molecular dynamics simulation of the CK2 crystal structure (portion data bank, PDB: 3C13) complexed with emodin was run for 100 nanoseconds (ns). The root-mean-square deviation (RMSD) of the protein changed from 0.8 Å at zero time to 4.50 Å at 100 ns; 0.8 Å to 1.8 Å and 0.4 Å to 0.8 Å for bikaverin, ellagic acid and emodin, respectively (Figure 9A). Ligand RMSDs (Figure 9B) demonstrate how stable the ligand is regarding the protein and its binding pocket. Figure 9B shows that the docking pose of bikaverin is rearranged in the beginning of the simulation, remaining stable for the rest of the time. Ellagic acid is also slightly rearranging after 16 ns and then stays stable for the rest of the simulation. As expected, redocking the originally co-crystallized emodin is the most stable compound in this test.

**Figure 10:**
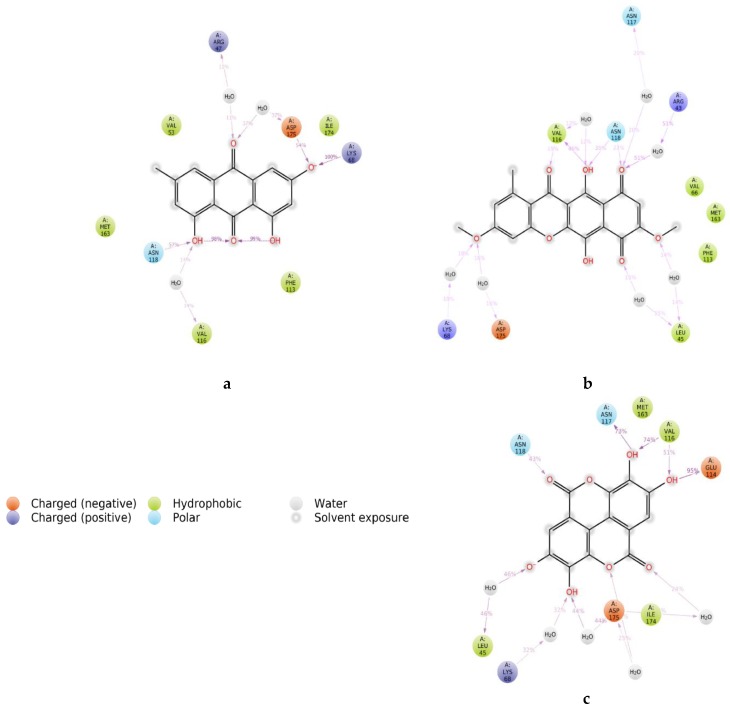
A schematic of detailed ligand atom interactions with the protein residues (**a**) emodin, (**b**) bikaverin and (**c**) ellagic acid.

**Figure 11:**
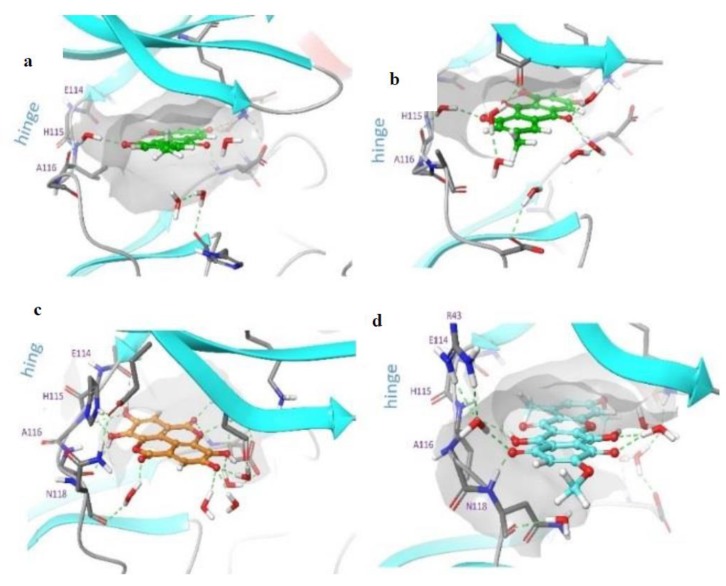
Snapshot structure of protein–ligand complexes at a time point of 100 ns. (**a**) presents an original PDB structure 1C13 (emodin), (**b**) emodin, (**c**) ellagic acid and (**d**) bikaverin. Only key interacting residues and the closest water molecules are shown for clarity. The orange dots represent negatively charged residues, violet dots represent the positively charged residues, green dots represent the hydrophobic regions, blue dots represent polar regions, and grey dots represent the water molecule.

Protein interactions with the ligand can be monitored throughout the simulation as well. These interactions can be categorized by type and were summarized as shown in Figure S1. Protein–ligand interactions (or “contacts”) are categorized into four types: hydrogen bonds, hydrophobic, ionic and water bridges. Each interaction type contains more specific subtypes, which can be explored through the “Simulation Interactions Diagram” panel. The stacked bar charts are normalized over the course of the trajectory: for example, a value of 0.7 suggests that in 70% of the simulation time, the specific interaction is maintained. Values over 1.0 are possible as some protein residues may make multiple contacts of the same subtype with the ligand. Figure 10 presents the “Ligand–Protein Contact Interactions” that occur more than 10–20% of the simulation time in the selected trajectory (0.00 through 100.00 ns). It is possible to have interactions with >100% as some residues may have multiple interactions of a single type with the same ligand atom. In Figure 11, we show the original X-ray structure and snapshot structures taken from the end of each simulation trajectory. The data reveals that in the case of emodin, the main contact is the salt bridge formed between its aromatic OH and lysine of the DFK motif. The contact to hinge main chain is supplied by bridging structural water. This contact remained stable throughout the simulations. According to the simulation data, bikaverin and ellagic acid may form direct hydrogen bond contact interactions within the hinge region.

Several studies have proven that protein kinase CK2 is involved in cell proliferation and survival and it is over-expressed in different human cancers [1,6]. In this work, we demonstrated that the natural compound bikaverin which is a potent CK2 inhibitor has a severe effect on different cancer cells. Actually, the current MTT results are in agreement with the results of Zhan et al. [29] in terms of MCF7 cells. It is important to mention that the cytotoxicity of bikaverin was also evaluated earlier using EAC, leukemia L5178 and sarcoma 37 cells, where it was suggested that bikaverin inhibits nucleic acid and protein synthesis [30]. Fuska et al. [30] tested some bikaverin derivatives in several cell lines as well [31], some of which were more effective than bikaverin. Unfortunately, none of these compounds were available to us now, but developing and testing some bikaverin derivatives is planned. In this study the effect of bikaverin on cell growth in three cancer cell lines was carried out using the live IncuCyte^®^ assay. The results showed that bikaverin has an anticancer effect at a concentration of 10 µM, and the cell morphology was changed over the test time. We also used the traditional cell viability assay (MTT assay) in parallel. Treatment with bikaverin altered the metabolism of the cells, which was confirmed by MTT assay as cell proliferation was reduced. The combination of the real time confluence monitoring (IncuCyte^®^ assay) and the end point cell variability assay (MTT assay) clearly indicates that bikaverin is more effective on MCF7 and A427 cells than A431, which might be related to the origin of these cells and their susceptibility to the inhibitor. Moreover, bikaverin was able to reduce the proliferation of the three cells dramatically as shown by EdU assay. The ability of a compound to enter the cells is a pivotal matter in drug discovery and development. Here we quantified first the cell membrane permeability of the three CK2 inhibitors (bikaverin, emodin and ellagic acid) by measuring their apparent permeability coefficient P_app_ using human epithelial colorectal adenocarcinoma cells (Caco-2 assay) [32,33]. It was possible to prove that bikaverin can penetrate the cell membrane very well. Finally, the molecular dynamics study suggested that bikaverin is stable in the ATP binding site of the enzyme which further explains its activity. In this work, we used a combination of in vitro evaluation, molecular dynamics simulations and molecular docking to understand the effect of bikaverin on the CK2 enzyme. The inhibitory activity of bikaverin using the holoenzyme is in the same micromolar range of emodin. Nevertheless, the better penetration of bikaverin can give this compound advantage over emodin as a promising natural hit which was also confirmed by the current cellular assay. Our data demonstrates that bikaverin is not only an active inhibitor of CK2 in vitro, but also has a dramatic effect on cell viability and proliferation in different cancer cell lines. It is important to note that the current results were obtained using a low dose of maximum 10 µM of the inhibitor; more significant effects could be obtained with higher doses. Taken together, the inhibitory effect of bikaverin as well as its cellular effects, its good cell permeability, and its natural origin can make this compound an interesting candidate for further development as an anti-cancer compound.

## 3. Materials and Methods

### 3.1. Compounds

Bikaverin and ellagic acid were purchased from Sigma-Aldrich (Darmstadt, Germany). Emodin was purchased from Merck Millipore (Darmstadt, Germany). All commercial reagents were of the highest available purity grade. The compounds were dissolved in dimethyl sulfoxide (DMSO) and the stock solutions were stored at −20 °C and warmed to 25 °C just before use.

### 3.2. Caco-2 Cell Permeability Assay

Human epithelial colorectal adenocarcinoma cells (Caco-2) were used to elucidate membrane permeability of bikaverin. Caco-2 cells were cultured using Dulbecco’s modified Eagle’s medium (DMEM) with the addition of 1% (*v*/*v*) non-essential amino acids, 1% (*v*/*v*) penicillin/ streptomycin/glutamine, and 10% fetal calf serum (FCS) in a humidified chamber at 5% CO_2_ and 37 °C. Cells were seeded at a density of 6 × 10^5^ cells/well on transwell filters (Transwell^®^, 12 well plate, Corning^®^, Corning, USA,) and were cultivated for three weeks. Medium was replaced three times a week. The integrity of the monolayer was determined using Trans Epithelial Electrical Resistance (TEER). For the permeability assay, medium in the apical compartment was removed and replaced by fresh medium containing bikaverin at a concentration of 50 µM, 100 µM emodin or 100 µM ellagic acid. The final concentration of DMSO was 1%. Rhodamine B (10 µg/mL) served as a control for a permeable substance (positive control) and FITC-dextran-4 (10 µg/mL) as a control for a non-permeable substance (negative control). Treated Caco-2 cells were incubated for 24 h at 37 °C in 5% CO_2_. The integrity of the monolayer was assessed by monitoring the TEER values every 20 min. After 24 h, the concentrations of CK2 inhibitors as well as control substances were determined in the basolateral compartment. Permeation of the control substances was evaluated by measuring the emitted fluorescence at 627 nm for Rhodamine B (excitation at 554 nm) and at 528 nm for FITC-dextran-4 (excitation at 485 nm). RP-HPLC (EC 125/4 Nucleodur C18 Htec, Macherey-Nagel, Düren, Germany) was used to measure the concentration of bikaverin as well as emodin and ellagic acid in the basolateral samples. Before separation, the samples were purified using solid phase extraction (Chromafix C_18_ec, Macherey-Nagel) following the procedure of Tóth et al. [34]. Purified samples (20 µL) were injected into the RP-HPLC. The chromatography was performed with a flow rate of 0.5 mL/min and a run time of 17 min at a temperature of 40 °C with UV monitoring at 250 nm and with a gradient mobile phase ranging from 10–90% (*v*/*v*) CH_3_CN in H_2_O with 0.05% trifluoro acetic acid. The apparent permeability coefficient (P_app_) was calculated according to the published equation [35]:

(1) $$P_{\mathrm{app}}{=V}_{b}\times \frac{c_{b}}{t}\times \frac{1}{c_{0}\times A}$$

where V_b_ is the volume of the recipient (basolateral) compartment, c_b_ is the concentration of the compound in the basolateral compartment, t is the incubation time, c_0_ is the initial concentration of the compound in the donor (apical) chamber and A is the membrane surface area.

### 3.3. Cell Culture and Proliferation

MCF7 human breast adenocarcinoma cells, provided by the Department of Clinical Radiology, Münster University Hospital, Germany, and A427 human lung carcinoma cell line purchased from DSMZ (Deutsche Sammlung von Mikroorganismen und Zellkulturen GmbH, Braunschweig, Germany no. ACC234), were cultured in RPMI 1640 medium GlutaMax (Life Technologies, Massachusetts, MA, USA) and 10% fetal calf serum [36]. The human epidermoid carcinoma cell A431 provided by the Department of Experimental Tumor Biology, Münster University, were cultured in DMEM high glucose medium supplemented with 2 mM l-Glutamine and 10% FCS. Cells were seeded at a density of 5.0 × 10^4^ cells per well into 24-well culture plates. After overnight incubation, the seeding medium was removed and replaced with fresh medium containing bikaverin at 0.1 μM, 1 μM, 5 μM or 10 μM. DMSO, at a final concentration of 1%, served as a control. Cells were incubated for 24 h or 48 h at 37 °C in a humidified atmosphere (5% CO_2_). Cell proliferation was quantified by the EdU-click assay (Baseclick BCK-EdU555-1; Baseclick GmbH, München, Germany): the nucleoside analog 5-ethynyl-2′-deoxyuridine is incorporated during active DNA synthesis, and the 5-TAMRA-PEG3-azide fluorophore, used for detection, is coupled by click reaction. Afterwards, the nuclear DNA was stained using the DNA fluorophore Hoechst 33342 for 30 min at room temperature in the dark. Finally, cells were washed and overlaid with PBS [28]. Cellular fluorescence was monitored with a Keyence BZ-9000 fluorescence microscope (Keyence Corporation, Osaka, Japan) with the “hard-coated“ TRITC filter (excitation 543/22 nm; emission 593/40 nm) for TAMRA detection and the “hard coated” DAPI BP filter (excitation 377/50 nm; emission 447/60 nm) for the detection of Hoechst stained cells. The number of cells exhibiting an active DNA synthesis (staining with fluorophore 5- TAMRA-PEG3-azide) and the total number of cells (Hoechst staining) were counted. The results were expressed as a percent ratio of proliferating cells versus the total number of untreated cells. The CK2 inhibitor was assayed in triplicate, and the experiments were repeated three times.

### 3.4. Cell Viability Assay

The effect of CK2 inhibitors on the viability of MCF7, A427 and A431 cells was evaluated using an MTT assay [37]. This assay is a colorimetric assay that measures the conversion of MTT into violet formazan which is produced by succinate dehydrogenase in the intact mitochondria in viable cells. For the MTT assay, cells were seeded in 96-well plates at a density of 1 × 10^5^ cells per well and incubated for 24 h or 48 h at 37 °C in a humidified atmosphere (5% CO_2_). After overnight incubation, the seeding medium was removed and replaced with fresh medium containing the inhibitor at 0.1 μM, 1 μM, 5 μM or 10 μM. DMSO, at a final concentration of 1%, served as a control. Afterwards, the MTT reagent (Sigma Aldrich, Darmstadt, Germany) was added at a final concentration of 0.5 mg/mL. After incubation for 2 h at 37 °C, medium was discarded and 200 µL DMSO was added for the solubilization of the formazan. After mixing, the absorption was determined at 570 nm with a reference wavelength of 630 nm using a microplate reader. CK2 inhibitors were assayed in triplicate, and the experiments were repeated three times.

### 3.5. IncuCyte^®^ Cytotoxicity Assay

The IncuCyte^®^ S3 Live-Cell Analysis System (Sartorius, Michigan, MI, USA) was used for kinetic monitoring of cell growth and to determine the cytotoxic effect of bikaverin in the three cell lines. This analysis system allows an automated in-incubator method of monitoring live cells. The concentration-dependent growth/inhibitory activity of the bikaverin was evaluated against the above used cells. Cells were seeded at a density of 7.5 × 10^3^ cells per well in 96-well black-walled plates and cells were incubated for 24 h or 48 h at 37 °C in a humidified atmosphere (5% CO_2_). After overnight incubation, seeding medium was removed and replaced with fresh medium containing the inhibitor at 0.1 μM, 1 μM, 5 μM or 10 μM or 1% DMSO as a control. Cell growth was monitored for 48 h. Growth curves were generated by the algorithm in the “2017A Rev2” software (Schrödinger, version 11.0.018; New York, NY, USA) from data points acquired during 2 h interval imaging. All samples were plated in quadruplicate.

### 3.6. Molecular Dynamics

#### 3.6.1. Computational Study

Computational work was performed on an Intel (R) Core (TM) processor 3.20 GHz using (Maestro Schrödinger version 11.0.018; New York, NY USA), and MD simulations were carried out using GPU nodes of the CSC Taito Supercluster [38].

#### 3.6.2. Protein Structure Preparation and Molecular Docking

Protein structure (PDB: 3C13, resolution 1.95 Å) was preprocessed for docking using the Protein Preparation Wizard from the Schrodinger suite [39]. Hydrogen atoms were added, bond orders were assigned and water beyond 5.00 Å from any hetero groups was removed. Finally, a restrained minimization using OPLS3 force field was carried out. Prior to docking, all crystal water except bridging between the ligand and main chain carbonyl of VAL116 at the hinge region were omitted. The grid box for molecular docking was centered based on the coordinates of the co-crystallized emodin. Schrodinger’s Glide was used [40] in standard precision (SP) to redock the co-crystallized ligand (emodin) with good accordance with experimental geometry. However, SP docking of bikaverin and ellagic acid results in poor convergence and less favorable ligand–protein interactions. As a consequence, an induced fit docking protocol was used to provide some additional flexibility to the side chains of the protein binding site, including the bridging structural water. When using the induced fit docking protocol, compounds were further docked by Induced Fit Docking (IFD), which treats the nearby residues within 5 Å of the binding ligand as flexible, using SP precision.

#### 3.6.3. Molecular Dynamics Simulation

Molecular dynamics simulations were run for bikaverin, ellagic acid and emodin docked to the CK2 crystal structure co-crystalized with emodin (PDB: 3C13, resolution 1.95 Å), respectively. The protein–ligand complexes for simulations were chosen from among the top ranked docking poses after visual inspection. Molecular simulations of 100 ns were carried out using Desmond software [41] using the OPLS3 force-field in the NPT ensemble (T = 300 K) at a constant pressure of 1 bar. In the beginning of each simulation, a default equilibration protocol of Desmond was used to relax the MD systems and to provide a smooth start. Energy and trajectory atomic coordinate data were recorded at intervals of 10 ns. In this study, molecular dynamics simulations were done at 300 K.

### 3.7. Statistical Analysis

All statistical analyses and statistical diagrams were generated with GraphPad Prism v5 software (GraphPad Software, San Diego, CA, USA).

## Figures and Tables

**Table 1:** Structures of the tested compounds with their IC_50_ values for human CK2 holoenzyme and cell permeability coefficients.

Name	Chemical Structure	IC_50_ µM	Permeability Coefficients (cm/s)
**Bikaverin**	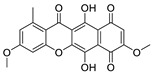	1.26 [9]	2.89 × 10^−6^
**Emodin**	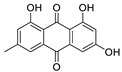	0.58 [24]	5.20 × 10^−6^
**Ellagic Acid**	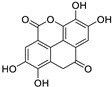	0.04 [24]	0.16 × 10^−6^

## Supplementary Materials

The following are available online, Figure S1: Protein interactions with (a) bikaverin, (b) ellagic acid, and (c) emodin.
