# Supplementary material for: In Vitro and In Silico Evaluation of Bikaverin as a Potent Inhibitor of Human Protein Kinase CK2
Source: Molecules. 2019 Apr 8;24(7):1380. doi: 10.3390/molecules24071380 (PMC6479664; doi:10.3390/molecules24071380)
Supplement: Supplementary file 1 [file molecules-24-01380-s001.pdf]

(a)

(b)

(c)

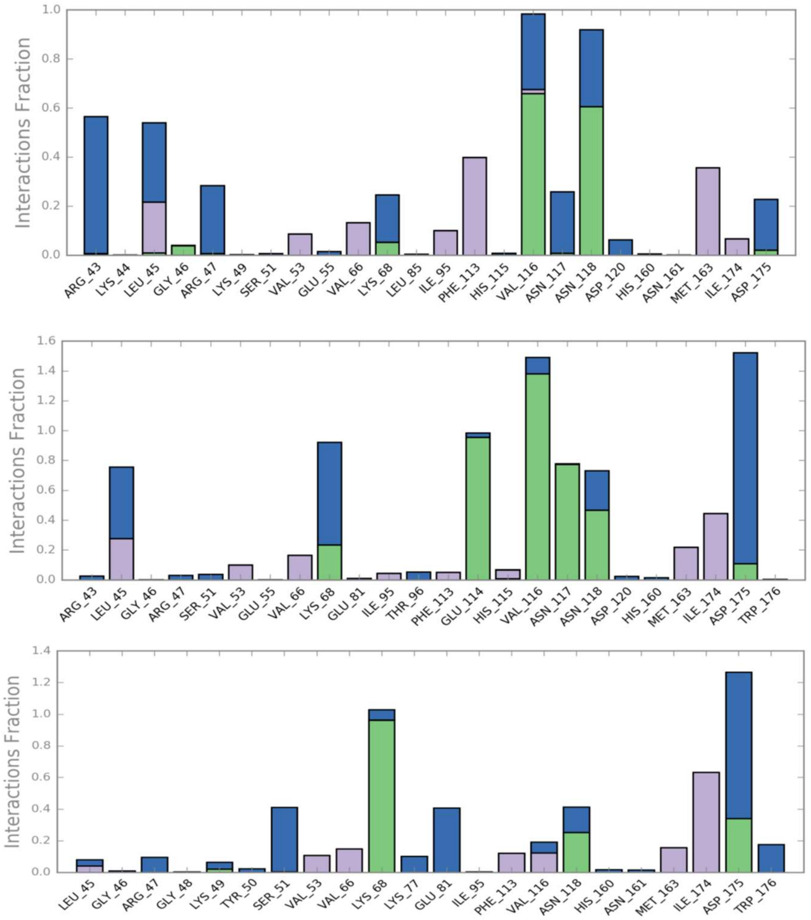

Figure S1. Protein interactions with (**a**) bikaverin, (**b**) ellagic acid, and (**c**) emodin. The green parts represent the hydrogen bonds, the violet parts represent hydrophobic, and blue parts represent water bridges.
